# Circular RNAs in gynecologic cancers: mechanisms and implications for chemotherapy resistance

**DOI:** 10.3389/fphar.2023.1194719

**Published:** 2023-06-08

**Authors:** Meiying Qin, Chunmei Zhang, Yang Li

**Affiliations:** Department of Obstetrics and Gynecology, Shengjing Hospital of China Medical University, Shenyang, Liaoning Province, China

**Keywords:** circular RNAs (circRNAs), gynecologic cancers, chemoresistance, malignant cancer, drug rsesistance

## Abstract

Chemotherapy resistance remains a major challenge in the treatment of gynecologic malignancies. Increasing evidence suggests that circular RNAs (circRNAs) play a significant role in conferring chemoresistance in these cancers. In this review, we summarize the current understanding of the mechanisms by which circRNAs regulate chemotherapy sensitivity and resistance in gynecologic malignancies. We also discuss the potential clinical implications of these findings and highlight areas for future research. CircRNAs are a novel class of RNA molecules that are characterized by their unique circular structure, which confers increased stability and resistance to degradation by exonucleases. Recent studies have shown that circRNAs can act as miRNA sponges, sequestering miRNAs and preventing them from binding to their target mRNAs. This can lead to upregulation of genes involved in drug resistance pathways, ultimately resulting in decreased sensitivity to chemotherapy. We discuss several specific examples of circRNAs that have been implicated in chemoresistance in gynecologic cancers, including cervical cancer, ovarian cancer, and endometrial cancer. We also highlight the potential clinical applications of circRNA-based biomarkers for predicting chemotherapy response and guiding treatment decisions. Overall, this review provides a comprehensive overview of the current state of knowledge regarding the role of circRNAs in chemotherapy resistance in gynecologic malignancies. By elucidating the underlying mechanisms by which circRNAs regulate drug sensitivity, this work has important implications for improving patient outcomes and developing more effective therapeutic strategies for these challenging cancers.

## 1 Introduction

**FIGURE 1:**
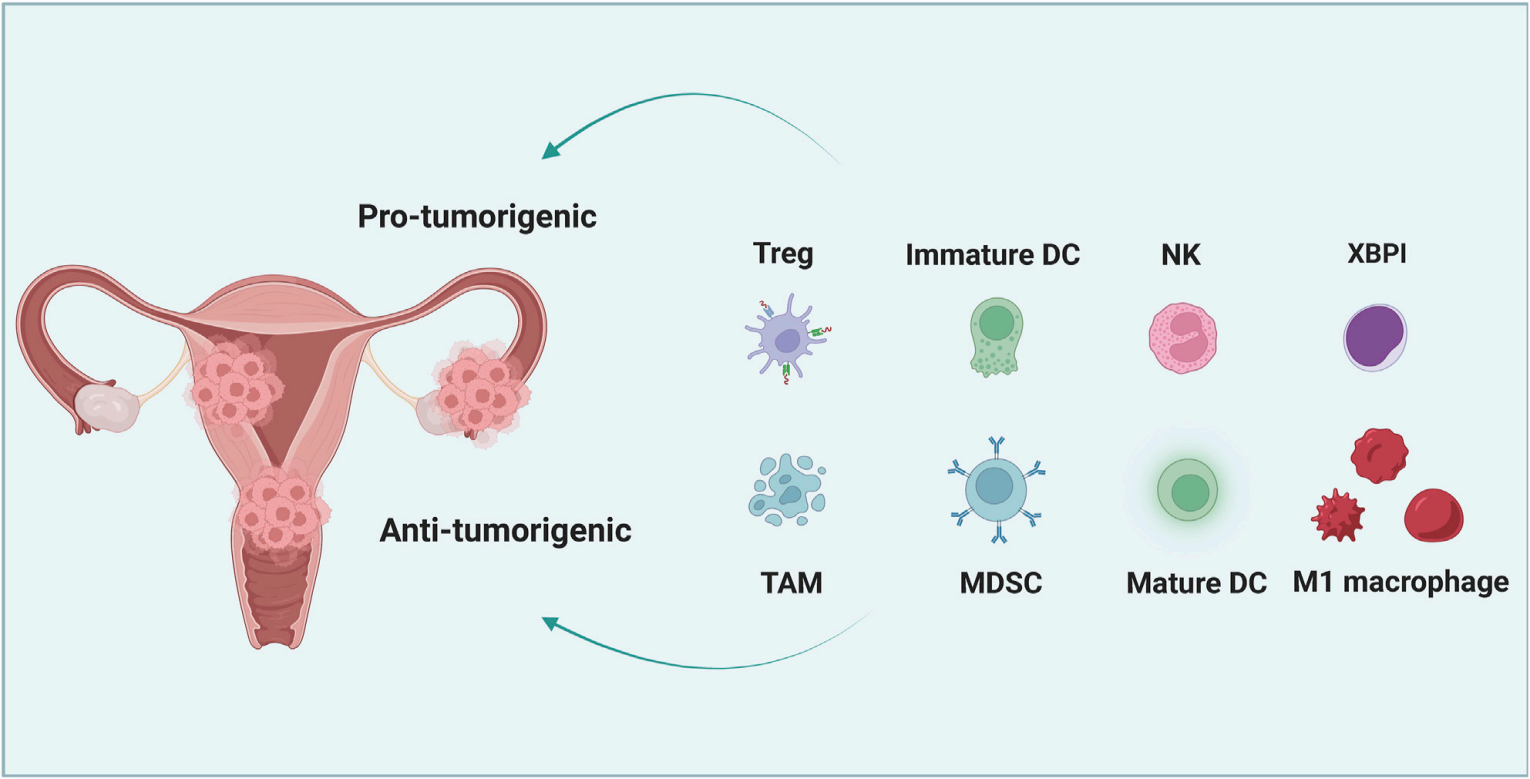
The interaction between cancer cells and immune cells in the microenvironment of gynecological cancer. Immature dendritic cells (DC), tumor-associated macrophages (TAM), regulatory T cells (Tregs) and myelogenous inhibitory cells (MDSCs) can promote the immune resistance and therapeutic resistance of gynecological cancer cells. However, mature DC, M1 macrophages, natural killer (NK) cells and cytotoxic T lymphocytes (CTL) can significantly inhibit tumor growth and increase the susceptibility of tumor cells to treatment.

The increasing incidence of gynecological tumors poses a significant concern, particularly in the cases of cervical cancer (CC), ovarian cancer (OC) and endometrial cancer (EC), which are considered widespread malignancies and gravely threaten women’s health (Diaz-Padilla et al., 2012; Lõhmussaar et al., 2020). Malignant gynecologic cancer is a significant contributor to the global burden of disease, accounting for three out of every ten deaths. As expected, cancer exerts a substantial impact on the economy, with the direct costs of cancer-related medical care in Australia amounting to approximately 0.5% of the country’s gross domestic product (GDP) (Goldsbury et al., 2018). Besides, the economic consequences of premature loss of life results in lost productivity valued at over $4 billion annually in Australia (Carter et al., 2016). Globally, cervical cancer is the fourth most prevalent malignancy, with an annual mortality of 270,000 individuals. This disease primarily impacts younger women, and its highest burden is observed in low- and middle-income countries, where the mortality rate is 18 times greater than in high-income countries (Sung et al., 2021). Ovarian cancer, on the other hand, is the seventh most common cancer among women worldwide, accounting for 3.3% of all female cancers. It is also the leading cause of death from gynecologic malignancies and the fifth highest among all cancers affecting women (Passarello et al., 2019). Variation in the incidence and mortality rates of ovarian cancer are observed worldwide, with the highest rates noted in developed countries such as Europe and North America (paragraph 3). Despite advancements in diagnosis and treatment, ovarian cancer continues to have a high case-fatality rate, with a 5-year survival rate of only approximately 30% for advanced-stage ovarian cancer (Webb and Jordan, 2017). Among these CC is primarily caused by persistent human papillomavirus (HPV) infection, with HPV types 16 and 18 responsible for 71% of cases worldwide (Choi et al., 2023; Reich and Regauer, 2023). Prevention and treatment of high-risk HPV cervical infections remain the main approach in combating CC, with the introduction of CC vaccines being a major development in recent years, together with screening technologies (Rahangdale et al., 2022; Rimel et al., 2022; Sivars et al., 2022; Sun et al., 2022; Sabeena, 2023). OC, as the seventh most commonly diagnosed female cancer worldwide, poses as the fifth leading cause of cancer-related deaths in women and the most lethal of all gynecological malignancies (Chen et al., 2023; Ye et al., 2023). Relatively few conventional screening tools exist for early detection, resulting in over 70% of the cases being diagnosed at advanced stages (Armbrister et al., 2023; Brown et al., 2023; Terp et al., 2023). The three main types of OC are epithelial, germ cell, and interstitial gonadal carcinoma, with epithelial carcinomas constituting the majority at about 90% of all OCs (Devlin and Miller, 2023; Zwimpfer et al., 2023). EC, on the other hand, is one of the most widespread malignancies occurring in the female reproductive tract, with inchoate phases typically being asymptomatic, while terminal phases feature symptoms akin to those of OC, including pelvic and abdominal pain, anemia, abdominal distention, wasting, and cachexia (Gordhandas et al., 2023). The current understanding of EC oncogenesis is still incipient, with most cases being sporadic and the few familial inherited cases resulting from mismatch repair protein gene mutations (Kalampokas et al., 2022; Tronconi et al., 2022). Predisposing risk factors for EC include obesity, infertility, and irregular menstrual cycles (Chiu et al., 2022; Jamieson and McAlpine, 2023). Furthermore, overexposure to endogenous or exogenous estrogens augments the risk of both endometrial hyperplasia and carcinogenesis, with conditions such as polycystic ovary syndrome, estrogen-secreting tumors, or the medical use of estrogen replacement therapy with inadequate progestin antagonism being implicated (Gjorgoska and Rizner, 2022; Yu et al., 2022). The tumor microenvironment plays a crucial role in modulating the malignant phenotype of various gynecological cancers, including enhancing their radiotherapy- and chemotherapy-tolerant properties, as well as their proliferative and metastatic potentials. Figure 1 illustrates the interaction between immune and cancer cells in the microenvironment of gynecological cancers. The currently available treatment of gynecologic tumors entails surgery, radiotherapy, and chemotherapy, there is a pressing need to explore alternative modalities that may yield more effective outcomes in the treatment of gynecologic tumors.

**FIGURE 2:**
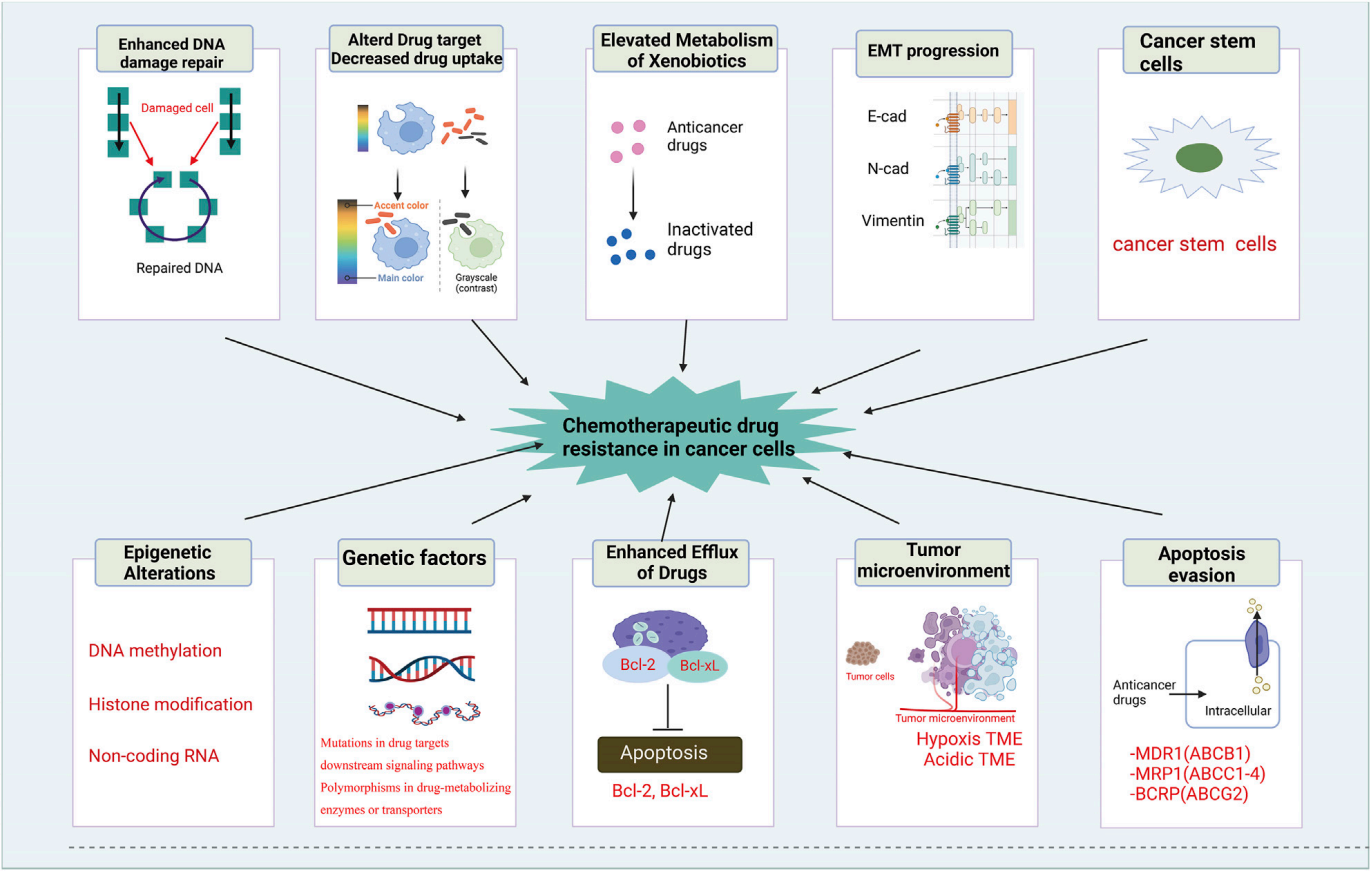
Mechanisms of chemotherapeutic drug resistance in cancer cells. The mechanisms of chemotherapeutic drug resistance in cancer cells includes enhanced DNA damage repair, altered drug target and decreased drug uptake, elevated metabolism of xenobiotics, EMT progression, cancer stem cells, epigenetic alterations, genetic factors, enhanced efflux of drugs, tumor environment and apoptosis evasion.

Significant advances in medical science have greatly improved anti-tumor therapy. However, drug resistance of tumor cells remains a major factor leading to high mortality rates (Gjorgoska and Rizner, 2022; Ming et al., 2023). Chemotherapy drug-sensitive tumors are present in only about 50% of cases, whereas acquired drug resistance is pervasive during treatment and a major contributor to chemotherapy failure (Liu et al., 2022a; Pang et al., 2023). Additionally, natural resistance of some tumor cells to multiple chemotherapeutic agents is prevalent, and drug resistance is estimated in no less than 90% of cancer deaths (Li et al., 2023a). Figure 2 describes the mechanisms of chemotherapeutic drug resistance in cancer cells. Although the mechanisms of drug resistance in gynecologic malignancies remain unknown, numerous studies have indicated a strong correlation between the development of gynecologic drug resistance and enhanced proliferation and migration of tumor cells, suppression of apoptosis, and immunosuppression (Alatise et al., 2022). Increasing evidence suggests that drug sensitivity in ovarian cancer (OC) is significantly influenced by non-coding RNAs (ncRNAs), tumor stem cells (CSCs), immune mechanisms, autophagy, and tumor heterogeneity (Cen et al., 2023; Tau and Miller, 2023). Additionally, it is evident that drug resistance in tumor cells is not solely dependent upon the sensitivity of individual tumor cells, but is tightly linked to the microenvironment in which the tumor cells reside (Li et al., 2022a; Parma et al., 2022). Further, the activation of given signaling pathways can regulate cell growth and differentiation, suppress apoptosis, and contribute to the development of drug resistance in tumor cells (Wang et al., 2022a; Yang et al., 2022a). The standard course of treatment for cervical, ovarian, and endometrial cancers is multifactorial and dependent upon several clinical criteria, including the stage, grade, and histologic type of the tumor, as well as the individual’s overall health and medical preferences. Treatment modalities generally entail surgical intervention, radiation therapy, and chemotherapy, typically administered in varying combinations. Surgery and radiation therapy represent the primary therapeutic options for cervical cancer, and chemotherapy may be given concurrently with radiation. Drug regimens currently recommended for cervical cancer may consist of cisplatin, paclitaxel, and carboplatin, among others. Ovarian cancer typically requires debulking surgery followed by chemotherapy. Chemotherapy for ovarian cancer generally involves a combination of agents, such as carboplatin and paclitaxel, delivered via intravenous or intraperitoneal routes. In the case of endometrial cancer, surgical resection is the mainstay of management, with chemotherapy reserved for advanced or recurrent disease. Standard chemotherapy regimens for endometrial cancer may incorporate drugs such as paclitaxel and carboplatin (Armstrong et al., 2021). It is essential to recognize that these treatments are not prescriptive and must be individualized based on patient and disease-specific features. Collaboration between the patient, medical oncologist, and gynecologic oncologist is crucial for determining appropriate therapeutic interventions. The choice of chemotherapy agents is ultimately influenced by the discretion of the treating physician, patient preference, and individual case intricacies.

**FIGURE 3:**
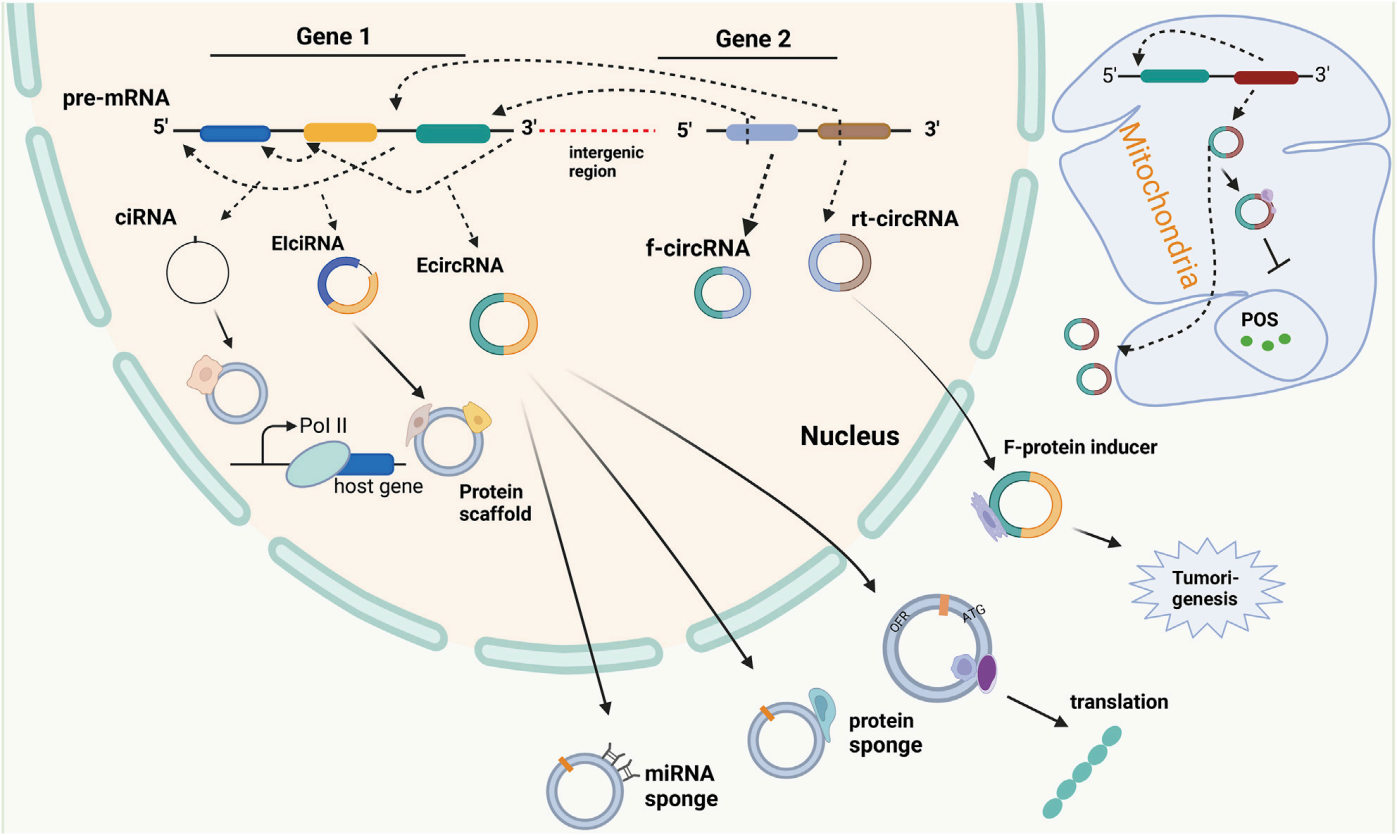
The biogenesis and function of circular RNA. Circular RNA (circRNA) is the product of reverse splicing of pre-messenger RNA (pre-mRNA), mainly including intron circRNA (ciRNA) from intron, exon and intron cirRNA (EIciRNA) from exon covering intron region, and exon circRNA (EcircRNA) from exon gene in nucleus and mitochondrial genome (MecciRNA). In addition, it also includes reading circRNA (rt-cicRNA) from the exon between adjacent genes on the same chain, and fusing circRNA (f-circRNA) from the exon between two distant genes. CircRNAs from different sources have different functions. CiRNA can interact with small ribonucleoprotein (snRNP) to improve the transcription rate of its host gene. EIciRNA can be used as a scaffold for recruiting functional molecules. ECIRcRNA can combine microRNAs and proteins to regulate the expression of downstream genes, and can also be used as a template for translation into new proteins and output to the cytoplasm. In addition, MecciRNA may be related to the inhibition of ROS. The combination of F-circRNA and fusion protein promotes tumorigenesis.

The circRNAs are a type of small RNA molecules characterized by their closed-loop structure that is formed by the exon skipping or reverse splicing of pre-mRNA transcripts, rendering them resistant to enzymatic degradation and thus highly stable within living organisms (Lee et al., 2022; Ren et al., 2022). Initially, circRNAs were deemed to be non-functional within the human body; however, the advent of high-throughput sequencing techniques has identified their extensive presence in various organs and tissues of the body, where they play crucial biological roles (Yuan et al., 2022; Zhou et al., 2022). Multiple studies have proposed that circRNAs contribute to essential physiological processes, such as tumorigenesis and development, and are inextricably linked to cancer cell proliferation, invasiveness, and metastasis (Chen et al., 2022a; Kim et al., 2023). More recent studies have demonstrated that circRNAs can modulate and influence drug resistance in different ways. For example, CircRNA_0067717 has been shown to facilitate paclitaxel (PTX) resistance in nasopharyngeal carcinoma, acting as a scaffold for TRIM41 and p53 (Cheng et al., 2023), whereas CircPOFUT1 enhances malignant traits and chemoresistance related to autophagy by binding to miR-488-3p and activating the PLAG1-ATG12 axis in cancer cells (Luo et al., 2023). CircPTK2 promotes epithelial-mesenchymal transition (EMT)-mediated bladder cancer metastasis and gemcitabine resistance by regulating the PABPC1/SETDB1 axis (Meng et al., 2023). To provide new insights into the management of drug resistance in gynecologic malignancies, this paper reviews the role and underlying mechanisms of circRNAs in chemoresistance in such cancers. CircRNAs were first detected in viruses in the 1970s, and at the time, due to limited understanding of circRNAs, they were thought to be splicing errors. The biogenesis and functions of circRNAs are demonstrated in Figure 3.

### 1.1 The biogenesis of circRNAs

CircRNAs are a unique class of RNA molecules generated from mRNA splicing events. Depending on their origin, CircRNAs are classified into three categories: exon-derived CircRNA (EcirRNA), intron-derived CircRNA (ciRNA), and exon- and intron-derived CircRNA (EIciRNA) (Caba et al., 2021; Huang and Zhu, 2021; Chen et al., 2022a; Liu et al., 2022b; Gao et al., 2022; Nielsen et al., 2022). Intron removal, a necessary step in mRNA splicing, leads to the formation of multiple mature mRNAs, each containing a unique combination of exons. The splicing complex mediates the nucleophilic site by using a branching site 2′-OH adenosine residues located between 20–50 nucleotides, leading to the formation of a lariat structure. This process involves the 3′ end of the upstream exon engaging in a nucleophilic attack on the 5′ splice site, resulting in the fusion of two exonic regions by breaking the phosphodiester backbone of the RNA molecule. In contrast to conventional splicing, the circularization of RNA can result from a process known as trans-splicing, though the exact mechanism is still under investigation. Two hypotheses have been proposed to explain the formation of CircRNAs by trans-splicing. The exon-skipping hypothesis suggests that two joining events are required to form the circular RNA structure, while in the direct trans-splicing hypothesis only one splicing event is involved in joining the 2′-OH branching point and the donor site of the intron^119^. The free 3′OH of the exon is then hypothesized to be responsible for the looping process leading to the formation of a closed looped structure.

### 1.2 Biological functions of circRNAs

CircRNAs refer to a class of RNA molecules that are generated through non-canonical splicing such as back-splicing or exon skipping of pre-mRNAs. These processes result in the formation of a continuous closed loop structure known as back-splicing, which is primarily induced via the junction of a downstream 3′ splice site with an upstream 5′ splice site (head-to-tail splicing) resulting in resistance of these molecules to exonucleolytic degradation by RNase R. Exon skipping can also lead to a restricted lariat structure promoting cyclization. Direct back-splicing often results in the generation of exonic circRNA (ecircRNA), while exon-skipping generates intronic circRNA. Currently, there are four categories of circRNAs, namely, ecircRNAs, circular intronic RNAs (ciRNAs), exon–intron circRNAs (EIciRNAs), and tRNA intronic circular RNAs (tricRNAs). ecircRNAs constitute over 80% of the identified circRNAs and are primarily located in the cytoplasm. ciRNAs and EIciRNAs, on the other hand, are predominantly located in the nucleus, suggesting a potential role in the regulation of gene transcription. Recently, a novel type of circular transcript called the read-through circRNA has been identified, which is formed through back-splicing of exons flanking a gene (Geng et al., 2020). According to recent studies, circRNAs are involved in pathophysiological processes *in vivo* through various mechanisms (Wang et al., 2022b). One of their more pervasive functions is that they can competitively bind microRNAs (miRNAs) and thus affect pathological processes such as tumor proliferation, aggression, and metastasis (Cheng et al., 2021; Zhou et al., 2021) (Zhang et al., 2021). Additionally, circRNAs can sponge-bind proteins, which may alter the transcription of parental genes, change the subcellular localization of proteins, and enable the interaction of multiple proteins among other effects (Zhou et al., 2020; Wu et al., 2021a; Das et al., 2021; Xu et al., 2022a). Interestingly, some circRNAs possess Internal Ribosome Entry Site (IRES) activity and open reading frame (ORF), which enable their translation into proteins *in vitro* or in cells (Sinha et al., 2021; Wen et al., 2022), (Yang et al., 2022a). Moreover, studies have demonstrated that elciRNA and ciRNA can adjust and control the transcriptional activity of RNA polymerase II (Pol II) and other transcription factors, which in turn regulate the expression of parental genes (Kim et al., 2021; Shao et al., 2021; Tang and Lv, 2021). Of course, additional regulatory mechanisms for circrna may require further investigation.

## 2 Circular RNAs and gynecologic cancer chemoresistance

### 2.1 CircRNA regulates cisplatin resistance in gynecologic cancer cells

Cisplatin (CDDP) is a commonly employed first-line treatment for gynecologic cancer. However, despite its effectiveness over years, repeated rejection of cis-CDDP frequently results in the death of these patients. Initially, CDDP was believed to interfere with DNA repair mechanisms by cross-linking with purine bases on DNA, leading to DNA damage and triggering apoptosis in cancer cells (Barman et al., 2023; Li et al., 2023b; Wang et al., 2023). Recent studies have revealed that CDDP also has harmful effects on various elements of the cell membrane and cytoplasm. Nonetheless, prolonged CDDP exposure leads tumor cells to activate a variety of mechanisms to obstruct cisplatin, which is manifested at the molecular, organelle, and cellular levels (Lugones et al., 2022; Romani, 2022; Tang et al., 2023). These mechanisms involve reducing platinum compound accumulation through active efflux/isolation or suppression of endocytosis; increasing oncogene mutagenesis; detoxifying through metallothionein, GSH conjugates, and other antioxidants; modulating DNA methylation status; increasing DNA-damage repair levels; altering protein post-translational modifications; over-expressing chaperone molecules; reinforcing compensatory signaling communication between organelles; suppressing apoptotic pathways; and activating the EMT pathway, among others (Ali et al., 2022; Domingo et al., 2022; Tsvetkova and Ivanova, 2022). Numerous studies have now demonstrated that certain circular RNAs (circRNAs) are also involved in drug resistance of gynecologic cancer cells to CDDP (Table 1). In particular, circEPSTI1 expression was significantly increased in both tissues and cells of cervical cancer (CC). Suppression of circEPSTI1 decreased the proliferative capability of CC cells and increased the sensitivity to cisplatin. Mechanistic experiments revealed that circEPSTI1 contributes to the malignant progression of CC by modulating the miR-370-3p-MSH2 axis, thereby leading to cisplatin resistance in CC (Wu et al., 2022). Similarly, studies have reported that the expression of circ-Cdr1as is significantly decreased in CDDP-resistant ovarian cancer (OC) tissues and cells. Overexpression of Cdr1as suppresses OC cell proliferation and promotes CDDP-induced apoptosis by modulating the miR-1270/SCAI signaling pathway (Zhao et al., 2019). Also, circHIPK2 expression was identified to be increased in CDDP-resistant OC tissues and cells. Suppression of circHIPK2 significantly suppressed the proliferation, cell cycle, migration, and invasion of SKOV3/CDDP and A2780/CDDP cells and promoted apoptosis. Mechanistic experiments showed that silencing circHIPK2 can regulate the miR-338-3p/CHTOP axis to suppress DDP resistance and malignant progression of OC (Cao et al., 2021). Compared to CDDP-sensitive OC cells, CDR1as expression was significantly reduced in CDDP-resistant OC cells. The downregulated expression of CDR1as suppressed OC tumorigenesis and predicted CDDP resistance and a poor prognosis in OC patients. Additionally, tumor xenograft data indicated that knockdown of CDR1as increased tumor growth and enhanced cell resistance to CDDP treatment (Wu et al., 2021b). CDR1as, also known as ciRS-7 (circular RNA sponge for miR-7), is a circular RNA molecule that has been shown to be involved in the pathogenesis of various cancers, including gynecologic malignancies such as endometrial cancer and ovarian cancer. CDR1as, also known as ciRS-7 (circular RNA sponge for miR-7), is a circular RNA molecule that has been shown to be involved in the pathogenesis of various cancers, including gynecologic malignancies such as cervical cancer and ovarian cancer. CDR1as upregulation was observed after TGF-β activation, which was positively correlated with lymph node metastasis and reduced survival duration, as evidenced by *in situ* hybridization. Overexpression of CDR1as was found to enhance cervical cancer metastasis both *in vitro* and *in vivo*. Furthermore, CDR1as was found to promote the orchestration of IGF2BP1 on the SLUG mRNA and to maintain its stability, thereby contributing to cervical cancer metastasis. Silencing IGF2BP1 hindered CDR1as-mediated metastasis in cervical cancer. Finally, it was found that CDR1as could activate TGF-β signaling factors, including P-Smad2 and P-Smad3, which promote EMT, demonstrating its potential role in EMT-related pathological processes (Zhong et al., 2023). The expression of CDR1as in ovarian tissues showed a significant difference between ovarian cancer patients and non-cancer controls, where the former exhibited lower levels of CDR1as expression. Overexpression of CDR1as significantly impeded the proliferation, invasion, and migration of ovarian cancer cells. In contrast, knockdown of CDR1as resulted in increased expression of miR-135b-5p and decreased levels of HIF1AN expression, ultimately elevating the proliferative potential of ovarian cancer cells (Chen et al., 2019a). Results of mechanistic experiments showed that CDR1as contributes to malignant progression of OC and CDDP resistance by regulating the miR-1299/PPP1R12B axis (Wu et al., 2021b). Additionally, it was found that circ_0063804 expression was remarkably upregulated in OC patients and predicts a poor prognosis. The overexpression of circ_0063804 in OC cells heightened resistance to cisplatin and decreased apoptosis. Results indicated that circ_0063804 can increase clusterin expression and thus lead to malignant phenotype and resistance to cisplatin in OC by sponging miR-1276 (You et al., 2022). Similarly, TYMP1 expression was also remarkably increased in OC tissues. Circ-TYMP1 functions as a sponge for miR-182A-3p and thus improves TGF1B expression, promoting proliferation, migration, aggression, and cisplatin resistance in A2780-Res cells and reducing Smad2/3 phosphorylation (Rao et al., 2022). Furthermore, circ_0026123 expression was increased significantly in both CDDP-resistant OC tissues and cells. Inhibition of circ_0026123 led to decreased cell growth, angiogenesis, invasion, and migration. It significantly increased the sensitivity of CDDP-resistive OC cells to CDDP, showing circ_0026123 could act as a sponge for miR-543 and thus increase the expression of RAB1A, thereby contributing to CDDP resistance and tumorigenesis in OC (Wei et al., 2022). Lastly, circ-PIP5K1A was highly expressed in CDDP-resistant OC tissues and cells. Suppression of circ-PIP5K1A restrained proliferation, migration, and invasion of CDDP-resistant OC cells, increased apoptosis, and sensitivity to CDDP. Mechanistically, circ-PIP5K1A could serve as a sponge for miR-942-5p and thus facilitate NFIB expression (Sheng and Wang, 2023). Sun et al. (2019), demonstrated a significant association between circPIP5K1A and the progression of ovarian cancer through its interaction with the miR-661/IGFBP5 axis. Silencing circPIP5K1A resulted in a downregulation of IGFBP5 due to an increase in miR-661 levels, which revealed that overexpression of IGFBP5 efficiently reversed the circPIP5K1A depletion effects. The conglomeration of these results suggests that circPIP5K1A is implicated in ovarian cancer’s progression by affecting the miR-661/IGFBP5 axis, and therefore, it may represent a viable target for therapeutic intervention of the disease (Sun et al., 2019). CircMTO1 expression was conspicuously increased in CC tissues and cell lines. It could improve migration, aggression, and CDDP resistance in CC cells and restrain apoptosis by regulating the miR-6893/S100A1/Beclin1/p62 signaling axis (Chen et al., 2019b).

**TABLE 1:** Potential roles of circRNAs in the cisplatin-resistance of gynecologic cancer.

Cancer	CircRNAs	Expression	Biological function	Targets	References
Cervical cancer	CircEPSTI1	Up	Promote cell proliferation and cisplatin resistance	miR-370-3p-MSH2	Wu et al. (2022)
	CircMTO1	Up	Promote cisplatin resistance and malignant progression	miR-6893/S100A1/Beclin1/p62	Chen et al. (2019b)
	CircARHGAP5	Down	Inhibit cell proliferation and cisplatin resistance, and promote cell apoptosis	AUF1/BIM	Deng et al. (2023)
	Hsa_circ_0023404	Up	Promote cell invasion, lymphatic formation and cisplatin resistance	miR-5047/VEGFA	Guo et al. (2019a)
				Beclin1/p62	
	Circ_0074269	Up	Promote cisplatin resistance and malignant progression	miR-485-5p/TUFT1	Chen et al. (2022b)
Ovarian cancer	Circ-Cdr1as	Down	Inhibit cell proliferation and cisplatin resistance, and promote cell apoptosis	miR-1270/SCAI	Zhao et al. (2019)
	CircHIPK2	Up	Promote cisplatin resistance and malignant progression	miR-338-3p/CHTOP	Cao et al. (2021)
	Circ-Cdr1as	Up	Promote cisplatin resistance and malignant progression	miR-1299/PPP1R12B	Wu et al. (2021b)
	circ_0063804	Up	Promote cell proliferation and cisplatin resistance, and inhibit cell apoptosis	miR-1276/CLU	You et al. (2022)
	Circ-TYMP1	Up	Promote cell proliferation, invasion and cisplatin resistance	miR-182A-3p/TGF1B/Smad2/3	Rao et al. (2022)
	Circ_0026123	Up	Promote cisplatin resistance and malignant progression	miR-543/RAB1A	Wei et al. (2022)
	Circ-PIP5K1A	Up	Promote cisplatin resistance and malignant progression	miR-942-5p/NFIB	Sheng and Wang (2023)
	CircITGB6	Up	Promote cisplatin resistance and induce polarization of TAMs towards M2 phenotype	IGF2BP2/FGF9	Li et al. (2022b)
	CircPBX3	Up	Promote cell colony formation and tumor growth and reduce cell apoptosis under cisplatin treatment	IGF2BP2/ATP7A	Fu et al. (2022)
	CircFoxp1	Up	Promote cell proliferation and cisplatin resistance	miR-22-miR-150-3p/CEBPG-FMNL3	Luo and Gui (2020)

In addition to their function as ceRNAs in regulating downstream gene expression, certain circular RNAs (circRNAs) have been demonstrated to regulate resistance to cisplatin (CDDP) in several ways including through protein binding and direct regulation of gene expression (as demonstrated in Table 1). For instance, the expression of circARHGAP5 is reduced in cervical squamous cell carcinoma (CSCC) tissues and overexpression of circARHGAP5 was found to hinder cisplatin-induced apoptosis in CSCC cells, ultimately leading to the progression of CSCC. Mechanistically, experiments indicated that under direct binding conditions, circARHGAP5 can inhibit the interaction between AUF1 and BIM mRNA, which enhances cisplatin resistance and the malignant transformation of CSCC (Deng et al., 2023). Similarly, it was reported that the expression of circITGB6 is conspicuously increased in tissues and sera of CDDP-resistant ovarian cancer (OC) patients and predicts poor prognosis. Overexpression of circITGB6 was found to promote M2 macrophage-dependent resistance to CDDP. Mechanistically, circITGB6 can directly interact with IGF2BP2 and FGF9 mRNA to form circITGB6/IGF2BP2/FGF9 RNA-protein ternary complexes in the cytoplasm, leading to increased stability of FGF9 mRNA and thereby inducing TAMs to polarize toward the M2 phenotype (Li et al., 2022b). Additionally, the expression of circPBX3 was significantly increased in both OC tissues and cisplatin-resistant OC cells, and overexpression of circPBX3 strongly promoted OC cell colony formation, tumor xenograft growth, and decreased apoptosis under cisplatin treatment. Mechanistic experiments suggested that circPBX3 can interact with IGF2BP2 to increase the stability of ATP7A mRNA and strengthen the level of ATP7A protein (Fu et al., 2022). Similarly, hsa_circ_0023404 was shown to be significantly increased in cervical cancer (CC) and its overexpression was found to facilitate VEGFA expression by binding miR-5047 and resulting in increased aggression of CC cells and lymphatic vessel formation in HDLEC cells. Furthermore, this circRNA also regulates the expression of autophagy-related genes (Beclin1 and p62), improving cisplatin resistance in CC cells (Guo et al., 2019a).

Moreover, it has been demonstrated that some circRNAs present in exosomes are also involved in regulating CDDP resistance (as outlined in Table 1). For example, circ-PIP5K1A is highly expressed in CDDP-resistant OC tissues and cells, and its inhibition results in the inhibition of proliferation, migration, and aggression of CDDP-resistant OC cells, as well as an increase in apoptosis and susceptibility to CDDP. The underlying mechanism involves circ-PIP5K1A acting as a sponge for miR-942-5p, which facilitates NFIB expression. Additionally, circ-PIP5K1A can be packaged into exosomes and internalized by surrounding cells to mediate intercellular communication between OC cells (Sheng and Wang, 2023). Similarly, circ_0074269 is overexpressed in CDDP-resistant CC tissues and cells, and its silencing strengthens CDDP sensitivity, inhibiting proliferation, migration, and the induction of apoptosis in CDDP-resistant CC cells. Moreover, circ_0074269 is enriched in the exosomes of CDDP-resistant CC cells and can be transmitted between CC cells (Chen et al., 2022b). Finally, it was reported that circulating exosome circFoxp1 was significantly more highly expressed in epithelial ovarian cancer (EOC) patients, particularly those with CDDP resistance. High expression of circFoxp1 predicts a worse prognosis in EOC patients, and its overexpression in EOC cells promotes cell proliferation and confers CDDP resistance. Mechanistically, circFoxp1 positively regulates the expression of CCAAT enhancer binding protein gamma (CEBPG) and formin-like 3 (FMNL3) by binding miR-22 and miR-150-3p (Luo and Gui, 2020).

### 2.2 CircRNA regulates paclitaxel resistance in gynecologic cancer cell resistance

Paclitaxel (PTX), or tamsulosin, is a novel terpenoid compound that has been approved by the FDA for clinical use as an anti-leukemia and anti-tumor drug (Xu et al., 2022b; Smith et al., 2022). PTX exerts its antitumor effects by inducing and promoting microtubule polymerization, preventing depolymerization, suppressing spindle formation, and blocking mitosis (Zhao et al., 2022a; Rubinstein et al., 2022). While most patients with gynecologic cancer respond well to paclitaxel chemotherapy during their first treatment, paclitaxel resistance often occurs as the number of chemotherapy sessions increases (Ortiz et al., 2022). enhanced efflux of drugs by overexpression of drug efflux pumps, such as P-gp and MRP1 (Kamazawa et al., 2002), appears to be the major mechanism contributing to paclitaxel resistance in gynecologic cancers. While alterations in tubulin expression or stability, activation of prosurvival signaling pathways, and deregulation of mitotic checkpoints can all contribute to paclitaxel resistance, the overexpression of drug efflux pumps has been identified as a key contributor to resistance in paclitaxel-resistant ovarian and endometrial cancer cells (Guo et al., 2019b). Other mechanisms, such as altered drug target and decreased drug uptake, may also play a role in paclitaxel resistance, but the evidence suggests that enhanced efflux of drugs via overexpression of drug efflux pumps is the most prevalent mechanism. Drug resistance is a critical factor leading to the mortality of patients. Recent studies have shown that circular RNAs (circRNAs) play a crucial role in PTX resistance in patients with gynecologic cancer and can act as competitive endogenous RNAs (ceRNAs) by binding to miRNAs and regulating downstream target genes (Table 2). CircMYBL2 is upregulated in cervical cancer (CC) tissues and cells, particularly in PTX-resistant CC tissues and cells. Overexpression of circMYBL2 enhances PTX resistance in CC cells, resulting in CC tumor growth. Mechanistic experiments demonstrate that circMYBL2 facilitates epidermal growth factor receptor (EGFR) expression, leading to PTX resistance by binding to miR-665 (Dong et al., 2021). Circ-CEP128 is conspicuously overexpressed in both CC tissues and cells, and its silencing in CC cells suppresses cell growth, migration, and aggression and heightens paclitaxel sensitivity by regulating the miR-432-5p/MCL1 axis (Zhao et al., 2022b). In another study, circ_0004488 is significantly increased in paclitaxel-resistant CC cells and highly expressed in cancer stem cell (CSC)-rich CC cell line subpopulations. Knockdown of circ_0004488 reduces cell proliferation, invasion, and spheroid formation in CC cells, thereby suppressing paclitaxel sensitivity. The outcomes of mechanistic experiments suggest that circ_0004488 enhances MEX3C expression by binding miR-136, thereby leading to CC malignancy progression and PTX resistance (Yi et al., 2022a). In ovarian cancer (OC), circCELSR1 is highly expressed in OC tissues and correlates with PTX resistance. Additionally, its expression is higher in PTX-resistant OC cells compared to PTX-sensitive cells. Suppression of circCELSR1 heightens PTX-induced cytotoxicity in OC cells, restraining tumor growth and promoting apoptosis by regulating miR-1252-FOXR2 (Zhang et al., 2020). CircTNPO3 expression is remarkably higher in OC samples and correlates with PTX resistance. Suppression of circTNPO3 in OC cells promotes PTX-induced apoptosis and strengthens cellular sensitivity to PTX by binding to miR-1299 and facilitating the expression of NEK2 (Xia et al., 2020). Alternatively, the overexpression of circEXOC6B in OC cells inhibits OC proliferation and motility, reducing OC resistance to PTX. The mechanistic outcomes suggest that circEXOC6B upregulates forkhead box O3 (FOXO3) expression by sponging miR-376c-3p, leading to PTX sensitivity in OC cells (Zheng et al., 2020). Moreover, circNRIP1 is highly expressed in PTX-resistant OC tissues and cells. Its suppression in OC cells restricts PTX resistance by regulating the miR-211-5p/HOXC8 axis (Li et al., 2020). Similarly, circ_0061140 facilitates chromobox 2 (CBX2) expression by binding to miR-136, leading to malignant OC progression and PTX resistance (99). On the other hand, circSETDB1 regulates PTX resistance in OC cells by targeting the miR-508-3p/ABCC1 axis (Huang et al., 2023). In endometrial cancer (EC), circ_0007534 is highly expressed and associated with poor prognosis in EC patients. Overexpression of circ_0007534 in EC cells enhances cell proliferation, aggression, epithelial-mesenchymal transition (EMT), and PTX resistance. The outcomes of mechanistic experiments show that circ_0007534 promotes EC invasiveness, progression, and PTX resistance by sponging miR-625 and promoting zinc finger E-box binding homeobox 2 (ZEB2) expression (Yi et al., 2022b). In contrast, the knockdown of circ_0039569 in EC cells restrains cell growth and invasion, leading to PTX sensitivity. Mechanistically, circ_0039569 promotes PTX resistance in EC by binding to miR-1271-5p and regulating plant homeodomain finger protein 6 (PHF6) (Li et al., 2022c).

**TABLE 2:** Potential roles of circRNAs in the paclitaxel-resistance of gynecologic cancer.

Cancer	CircRNAs	Expression	Biological function	Targets	References
Cervical cancer	CircMYBL2	Up	Enhance PTX resistance and promote tumor growth	miR-665/EGFR	Dong et al. (2021)
	Circ-CEP128	Up	Promote cell growth, migration and invasion and inhibit PTX sensitivity	miR-432-5p/MCL1	Zhao et al. (2022b)
	Circ_0004488	Up	Promote cell proliferation, invasion, and spheroid formation and inhibits PTX sensitivity	miR-136/MEX3C	Yi et al. (2022a)
Ovarian cancer	CircCELSR1	Up	Enhance PTX resistance and promote tumor growth	miR-1252/FOXR2	Zhang et al. (2020)
	CircTNPO3	Up	Inhibit cell apoptosis and promote PTX resistance	miR-1299/NEK2	Xia et al. (2020)
	CircEXOC6B	Down	Inhibit cell proliferation and movement and reduce PTX resistance	miR-376c-3p/FOXO3	Zheng et al. (2020)
	CircNRIP1	Up	Enhance PTX resistance	miR-211-5p/HOXC8	Li et al. (2020)
	Hsa_circ_0000714	Up	Enhance PTX resistance and promote tumor growth	miR-370-3p/CDK6/RB/RAB17	Guo et al. (2020)
	Circ_CELSR1	Up	Enhance PTX resistance and promote tumor growth	miR-149-5p/SIK2	Wei et al. (2021)
	Circ_0061140	Up	Enhance PTX resistance and promote tumor growth	miR-136/CBX2	Zhu et al. (2021)
	CircSETDB1	Up	Enhance PTX resistance	miR-508-3p/ABCC1	Huang et al. (2023)
	CircANKRD17	Up	Promote cell viability, PTX resistance and inhibit cell apoptosis	FUS/FOXR2	Liang et al. (2022)
Endometrial cancer	Circ_0007534	Up	Promote cell proliferation, invasion, EMT and PTX resistance	miR-625/ZEB2	Yi et al. (2022b)
	Circ_0039569	Up	Promote cell growth and invasion and	miR-1271-5p/PHF6	Li et al. (2022c)

In addition to binding miRNAs to regulate downstream gene expression, some circRNAs also adjust and control PTX resistance by binding proteins (Table 3). CircANKRD17 is highly expressed and prognostic of poor outcomes in PTX-resistant OC tissues and cells. Its knockdown suppresses PTX resistance in OC cells by suppressing cell viability and inducing apoptosis. Mechanistically, circANKRD17 stabilizes forkhead box R2 (FOXR2) by interacting with fused in sarcoma (FUS), leading to PTX resistance in OC through the circANKRD17/FUS/FOXR2 signaling axis (Liang et al., 2022).

**TABLE 3:** Potential of chemoresistance related circRNAs as dianostic and prognostic tools in gynecologic cancer.

Cancer	CircRNA	Detection method	*p*-value	Diagnosis	FIGO (*p*-value)	LNM (*p*-value)	DM (*p*-value)	OS (*p*-value)	DFS (*p*-value)	Follow-up (months)	References
Ovarian camcer	CircTNPO3	Specific qRT-PCR	*p* < 0.001	AUC = 0.910	*p* = 0.008	*p* = 0.57	*p* = 0.082	*p* = 0.030	/	60	Xia et al. (2020)
	CircFoxp1	Specific qRT-PCR	*p* < 0.001	AUC = 0.914	*p* = 0.0312	*p* = 0.0009	*p* = 0.0394	*p* < 0.0001	*p* < 0.0001	60	Luo and Gui (2020)
	CircEXOC6B	Specific qRT-PCR	*p* < 0.05	/	*p* < 0.05	*p* < 0.05	/	*p* = 0.012	/	60	Zheng et al. (2020)
	CircITGB6	Specific qRT-PCR	*p* < 0.001	/	/	/	/	*p* = 0.006	*p* < 0.001	60	Li et al. (2022b)
	CircANKRD17	Specific qRT-PCR	*p* < 0.001	/	/	/	/	*p* = 0.033	/	60	Liang et al. (2022)
	CircSETDB1	Specific qRT-PCR	*p* < 0.001	/	/	/	/	*p* = 0.012	/	60	Huang et al. (2023)
	Circ_0063804	Specific qRT-PCR	*p* < 0.001	/	*p* < 0.05	/	*p* = 0.508	*p* = 0.0197	/	60	You et al. (2022)
	CircPBX3	Specific qRT-PCR	*p* < 0.001	/	*p* < 0.001	*p* = 0.010	*p* = 0.783	/	/	/	Fu et al. (2022)
Cervical cancer	Circ_0004488	Specific qRT-PCR	*p* < 0.001	/	/	/	/	*p* < 0.001	/	60	Yi et al. (2022a)
Endometrial cancer	Circ_0007534	Specific qRT-PCR	*p* < 0.001	/	*p* < 0.001	/	*p* < 0.001	*p* = 0.012	/	60	Yi et al. (2022b)

### 2.3 CircRNAs regulate resistance of gynecologic cancer cells to other chemotherapeutic agents

Several research studies have demonstrated that circular RNAs (circRNAs) have the potential to regulate the resistance of gynecologic cancer cells to other chemotherapeutic agents, as depicted in Table 3. Several research studies have demonstrated that circular RNAs (circRNAs) have the potential to regulate the resistance of gynecologic cancer cells to other chemotherapeutic agents, such as docetaxel (DTX), as depicted in Table 3. Treatment of SKOV3-R cells with DTX led to a significant decrease in the expression of circRNA_0006404, while an upregulation in circRNA_0000735 expression was observed. circRNA_0000735 was found targeted by miR-526b, which subsequently regulated the expression of DKK4 and p-GP, leading to chemotherapy resistance in SKOV3-R cells treated with DTX (Chen and Tai, 2022). Medroxyprogesterone acetate (MPA) constitutes one of the most commonly administered progesterone treatments for endometrial cancer (EC), whereas hsa_circ_0001860 expression was noted to be significantly decreased in MPA-resistant tissues and cells, with a negative correlation noted with lymph node metastasis and histological grading of EC. Observation of the downstream effects of inhibiting hsa_circ_0001860 in EC cells included a conspicuous promotion of cell proliferation, migration, invasion and a suppressed apoptosis. The results obtained from mechanistic experiments have established that hsa_circ_0001860 promotes the expression of Smad7 when it binds to miR-520h (Yuan et al., 2021).

## 3 The diagnostic and prognostic value of drug resistance-associated circRNAs in gynecologic cancer

Drug-resistant related circular RNAs (circRNAs) are valuable in the early diagnosis and prognostic assessment of gynecologic cancers (GC). Certain circRNAs have diagnostic significance in GC, such as circTNPO3 which is highly expressed in ovarian cancer (OC) tissues and significantly correlates with the terminal Federation of Gynecology and Obstetrics (FIGO) stage and histological type of OC patients (Xia et al., 2020). ROC curve analysis of samples ranging from normal ovarian tissues to paclitaxel (PTX)-sensitive OC tissues (*n* = 20) to PTX-resistant OC tissues (*n* = 28) showed that circTNPO3 effectively distinguishes between PTX-sensitive and PTX-resistant OC tissues with an area under the ROC curve (AUC) of 0.910. Furthermore, Kaplan-Meier survival curve analysis revealed that OC patients with low circTNPO3 expression experienced significantly longer overall survival than those with high circTNPO3 expression. Another circRNA, exosomal circFoxp1, displayed conspicuously higher expression in the serum of epithelial OC (EOC) patients, showing an AUC value of 0.914 in ROC curve analysis. Additionally, serum exosome circFoxp1 expression is associated with FIGO stage, primary tumor size, lymph node metastasis, distal metastasis, residual tumor diameter, clinical response, and histological type and grade. The aforementioned results suggest that exosomal circFoxp1 can serve as a valuable biomarker for EOC patients, as lower overall survival and disease-free survival were observed in patients with higher expression levels of circFoxp1 (Luo and Gui, 2020).

The study highlights the prognostic significance of the expression levels of some circRNAs in gynecological tumors. Specifically, in PTX-resistant cervical cancer (CC) tissues, it was found that the expression of circ_0004488 was remarkably higher than in PTX-sensitive CC tissues. Moreover, the Kaplan-Meier survival curves showed that increasing levels of circ_0004488 were associated with a decrease in overall survival of CC patients (Yi et al., 2022a). Similarly, in ovarian cancer (OC), the expression of circEXOC6B was observed to decrease and was negatively correlated with tumor progression. Furthermore, high expression of circEXOC6B was linked to long-term survival time in OC patients (Zheng et al., 2020). Conversely, in CDDP-resistant OC patients, the expression levels of circITGB6 were significantly upregulated as compared to those in CDDP-sensitive OC patients and normal controls. Notably, OC patients with high levels of circITGB6 had a relatively low overall survival rate and a higher relapse rate, as determined by survival analysis (Li et al., 2022b). Additionally, the expression of circANKRD17 was significantly upregulated in OC tissues, with patients with higher circANKRD17 expression demonstrating a shorter overall survival time compared to those with low expression (Liang et al., 2022). The expression of circSETDB1 was found to be notably higher in PTX-resistant ovarian cancer tissues than in normal tissues. Importantly, OC patients with high circSETDB1 expression had a worse prognosis, according to Kaplan-Meier survival curve analysis (Huang et al., 2023).

Furthermore, some circRNAs were found to be associated with clinical features of gynecologic cancer. For instance, circ_0007534 expression levels were significantly higher in endometrial cancer (EC) tissues, and high expression of circ_0007534 predicted worse tumor differentiation, more terminal pathological phase, deeper infiltration, and stronger cancer metastasis. Importantly, patients with high circ_0007534 expression level had a significantly shorter survival time (Yi et al., 2022b). Similarly, it was observed that in OC tumor tissues, the expression of circ_0063804 was remarkably higher than in normal control tissues. Additionally, high expression of circ_0063804 was strongly correlated with lower survival, terminal FIGO stage and grade, and larger tumor size, as determined by various analyses (You et al., 2022) Finally, the expression of circPBX3 was found to be highly upregulated in OC, and high expression of circPBX3 was positively correlated with larger tumor size, terminal FIGO stage, and lymph node metastasis, as determined by analysis (Fu et al., 2022).

## 4 Conclusion and perspective

Chemotherapy has long been considered one of the most effective treatments for cancer. Despite this, the development of drug resistance has proved to be a major obstacle to successful patient outcomes (Wang et al., 2022c; Karami Fath et al., 2022; Pastwińska et al., 2022). Chemotherapy exerts its cytotoxic effects by inhibiting cellular synthesis of DNA and RNA, suppressing cell proliferation, and promoting apoptosis (Abdelaal and Haffez, 2022; Yang et al., 2022b; Li et al., 2022d). However, the efficacy of chemotherapy is limited by drug resistance, which leads to tumor progression and ultimately patient mortality. Initial studies on drug resistance in tumors identified several protein-encoding genes that are closely associated with chemoresistance development, including the drug transport proteins MDR1, MRP, and ABCG2 (Chimento et al., 2022; Yang et al., 2022c; Zhao et al., 2022c; Vaghari-Tabari et al., 2022). Recent advances in molecular analysis and high-throughput sequencing techniques have enabled rapid and accurate identification of the expression profiles of non-coding RNAs associated with drug resistance (Sánchez-Marín et al., 2022). Due to the chemotherapy resistance and early-stage metastasis of gynecological cancer, the prognosis for patients is unfavorable, and the 5-year survival rate remains low despite aggressive treatment. Consequently, identifying reliable biomarkers and gaining insight into the molecular mechanisms of chemoresistance in gynecological cancer is critical to developing new anti-gynecological cancer strategies. High-throughput RNA sequencing has proven useful in identifying circRNAs that are dysregulated in association with gynecological cancer chemoresistance and elucidating their potential mechanisms. This paper presents the circRNAs associated with chemoresistance identified in the mentioned research, which are involved in the regulation of drug metabolism, DNA damage repair, apoptosis and EMT signaling pathways. Some of these circRNAs may even serve as valuable prognostic markers.

The search for circRNAs associated with drug resistance in gynecologic cancers has the potential to minimize the “experimental” use of drugs and enable more rational selection of treatment regimens. Furthermore, combining circRNA inhibitors or enhancers with chemotherapeutic drugs can enhance chemotherapy sensitivity. For patients who are dose-limited, adding circRNAs to targeted therapy, while decreasing the dose of chemotherapeutic drugs, could significantly reduce the adverse effects of dose limitation and alleviate the discomfort caused by treatment. Nonetheless, the development and clinical application of related circRNAs remain inadequate. Tumor drug resistance is a multifactorial trait, and the complexity of the tumor microenvironment may result in differences in *ex vivo* research. This complexity makes targeting circRNAs to enhance chemotherapy sensitivity challenging and uncertain.

Our manuscript provides a comprehensive review of the role of circular RNAs (circRNAs) in chemotherapy resistance in gynecologic malignancies and their mechanisms. While there have been some previous studies on this topic, our review offers several novel and innovative contributions to the literature. Firstly, we have identified specific circRNAs that are involved in regulating chemotherapy resistance for different chemotherapeutic agents used in the treatment of gynecologic malignancies. This information can be used to develop more targeted and effective treatment strategies. Secondly, we have discussed the mechanisms by which these circRNAs regulate chemotherapy resistance, including drug metabolism, DNA injury repair, apoptosis and EMT signaling pathways. By understanding these mechanisms, researchers and clinicians can develop new approaches to overcome drug resistance. Thirdly, we have highlighted the potential clinical applications of circRNAs as biomarkers for predicting chemotherapy response and as therapeutic targets for improving treatment outcomes in patients with gynecologic malignancies. Overall, our manuscript offers a unique perspective on the role of circRNAs in chemotherapy resistance in gynecologic malignancies and provides valuable insights into potential new approaches for improving treatment outcomes.

## Abbreviations

AUC, area under the ROC curve; Beclin1 and p62, autophagy-related genes; CEBPG, CCAAT enhancer binding protein γ; CC, cervical cancer; CDDP, Cisplatin; ciRNA, intron-derived circRNA; circRNA, Circular RNA; CSCC, cervical squamous cell carcinoma; CSCs, tumor stem cells; EC, Endometrial cancer; EC, endometrial cancer; EcirRNA, exon-derived circRNA; EIciRNA, exon- and intron-derived circRNA; FMNL3, formin like 3; HPVhuman, papillomavirus; IRES, Internal Ribosome Entry Site; miRNAs, microRNAs; MPA, Medroxyprogesterone acetate; ncRNAs, non-coding RNAs; OC, Ovarian cancer; ORF, open reading frame; Pol II, polymerase II; pre-mRNAs, precursor mRNAs; PTX, Paclitaxel.

## References

[B1] Abdelaal M. R. Haffez H. (2022). The potential roles of retinoids in combating drug resistance in cancer: Implications of ATP-binding cassette (ABC) transporters. Open Biol. 12 (6), 220001. 10.1098/rsob.220001 35642494 PMC9157304

[B2] Alatise K. L. Gardner S. Alexander-Bryant A. (2022). Mechanisms of drug resistance in ovarian cancer and associated gene targets. Cancers (Basel) 14 (24), 6246. 10.3390/cancers14246246 36551731 PMC9777152

[B3] Ali R. Balamurali M. Varamini P. (2022). Deep learning-based artificial intelligence to investigate targeted nanoparticles' uptake in TNBC cells. Int. J. Mol. Sci. 23 (13), 16070. 10.3390/ijms232416070 36555718 PMC9785476

[B4] Armbrister R. Ochoa L. Abbott K. L. (2023). The clinical role of glycobiology on ovarian cancer progression. Adv. Cancer Res. 157, 1–22. 10.1016/bs.acr.2022.07.004 36725106

[B5] Armstrong D. K. Alvarez R. D. Bakkum-Gamez J. N. Barroilhet L. Behbakht K. Berchuck A. (2021). Ovarian cancer, version 2.2020, NCCN clinical practice guidelines in oncology. J. Natl. Compr. Canc Netw. 19 (2), 191–226. 10.6004/jnccn.2021.0007 33545690

[B6] Barman R. Bej R. Dey P. Ghosh S. (2023). Cisplatin-conjugated polyurethane capsule for dual drug delivery to a cancer cell. ACS Appl. Mater Interfaces 15 (21), 25193–25200. 10.1021/acsami.2c22146 36745598

[B7] Brown Y. Hua S. Tanwar P. S. (2023). Extracellular matrix in high-grade serous ovarian cancer: Advances in understanding of carcinogenesis and cancer biology. Matrix Biol. 118, 16–46. 10.1016/j.matbio.2023.02.004 36781087

[B8] Caba L. Florea L. Gug C. Dimitriu D. C. Gorduza E. V. (2021). Circular RNA-is the circle perfect? Biomolecules 11 (12), 1755. 10.3390/biom11121755 34944400 PMC8698871

[B9] Cao Y. Xie X. Gao Y. (2021). CircHIPK2 contributes to DDP resistance and malignant behaviors of DDP-resistant ovarian cancer cells both *in vitro* and *in vivo* through circHIPK2/miR-338-3p/CHTOP ceRNA pathway. Onco Targets Ther. 14, 3151–3165. 10.2147/OTT.S291823 34012271 PMC8128508

[B10] Carter H. E. Schofield D. J. Shrestha R. (2016). The productivity costs of premature mortality due to cancer in Australia: Evidence from a microsimulation model. PLoS One 11 (12), 0167521. 10.1371/journal.pone.0167521 PMC5152930 27942032

[B11] Cen Y. Chen L. Liu Z. Lin Q. Fang X. Yao H. (2023). Novel roles of RNA-binding proteins in drug resistance of breast cancer: From molecular biology to targeting therapeutics. Cell. Death Discov. 9 (1), 52. 10.1038/s41420-023-01352-x 36759501 PMC9911762

[B12] Chen H. Mao M. Jiang J. Zhu D. Li P. (2019). Circular RNA CDR1as acts as a sponge of miR-135b-5p to suppress ovarian cancer progression. Onco Targets Ther. 12, 3869–3879. 10.2147/OTT.S207938 31190886 PMC6529026

[B13] Chen J. Wu S. Wang J. Sha Y. Ji Y. (2022). Hsa_circ_0074269-mediated upregulation of TUFT1 through miR-485-5p increases cisplatin resistance in cervical cancer. Reprod. Sci. 29 (8), 2236–2250. 10.1007/s43032-022-00855-9 35075616

[B14] Chen M. Ai G. Zhou J. Mao W. Li H. Guo J. (2019). circMTO1 promotes tumorigenesis and chemoresistance of cervical cancer via regulating miR-6893. Biomed. Pharmacother. 117, 109064. 10.1016/j.biopha.2019.109064 31226633

[B15] Chen Q. Li J. Shen P. Yuan H. Yin J. Ge W. (2022). Biological functions, mechanisms, and clinical significance of circular RNA in pancreatic cancer: A promising rising star. Cell. Biosci. 12 (1), 97. 10.1186/s13578-022-00833-3 35729650 PMC9210669

[B16] Chen Q. Shi J. Ruan D. Bian C. (2023). The diagnostic and therapeutic prospects of exosomes in ovarian cancer. Bjog. 10.1111/1471-0528.17446 36852533

[B17] Chen Y. Y. Tai Y. C. (2022). Hsa_circ_0006404 and hsa_circ_0000735 regulated ovarian cancer response to docetaxel treatment via regulating p-GP expression. Biochem. Genet. 60 (1), 395–414. 10.1007/s10528-021-10080-9 34255218

[B18] Cheng D. Wang J. Dong Z. Li X. (2021). Cancer-related circular RNA: Diverse biological functions. Cancer Cell. Int. 21 (1), 11. 10.1186/s12935-020-01703-z 33407501 PMC7789196

[B19] Cheng Y. Zhu Y. Xiao M. Zhang Y. Wang Z. Chen H. (2023). circRNA_0067717 promotes paclitaxel resistance in nasopharyngeal carcinoma by acting as a scaffold for TRIM41 and p53. Cell. Oncol. (Dordr) 46, 677–695. 10.1007/s13402-023-00776-y 36705889 PMC12974661

[B20] Chimento A. D'Amico M. Pezzi V. De Amicis F. (2022). Notch signaling in breast tumor microenvironment as mediator of drug resistance. Int. J. Mol. Sci. 23 (11), 6296. 10.3390/ijms23116296 35682974 PMC9181656

[B21] Chiu W. K. Kwok S. T. Wang Y. Luk H. M. Chan A. H. Y. Tse K. Y. (2022). Applications and safety of sentinel lymph node biopsy in endometrial cancer. J. Clin. Med. 11 (21), 6462. 10.3390/jcm11216462 36362690 PMC9658097

[B22] Choi S. Ismail A. Pappas-Gogos G. Boussios S. (2023). HPV and cervical cancer: A review of epidemiology and screening uptake in the UK. Pathogens 12 (2), 298. 10.3390/pathogens12020298 36839570 PMC9960303

[B23] Das A. Sinha T. Shyamal S. Panda A. C. (2021). Emerging role of circular RNA-protein interactions. Noncoding RNA 7 (3), 48. 10.3390/ncrna7030048 34449657 PMC8395946

[B24] Deng S. Qian L. Liu L. Liu H. Xu Z. Liu Y. (2023). Circular RNA ARHGAP5 inhibits cisplatin resistance in cervical squamous cell carcinoma by interacting with AUF1. Cancer Sci. 114, 1582–1595. 10.1111/cas.15723 36632741 PMC10067438

[B25] Devlin M. J. Miller R. E. (2023). Disparity in the era of personalized medicine for epithelial ovarian cancer. Ther. Adv. Med. Oncol. 15, 24. 10.1177/17588359221148024 PMC9837277 36643655

[B26] Diaz-Padilla I. Duran I. Clarke B. A. Oza A. M. (2012). Biologic rationale and clinical activity of mTOR inhibitors in gynecological cancer. Cancer Treat. Rev. 38 (6), 767–775. 10.1016/j.ctrv.2012.02.001 22381585

[B27] Domingo I. K. Latif A. Bhavsar A. P. (2022). Pro-inflammatory signalling PRRopels cisplatin-induced toxicity. Int. J. Mol. Sci. 23 (13), 7227. 10.3390/ijms23137227 35806229 PMC9266867

[B28] Dong M. Xie Y. Wang Z. Wang R. (2021). CircMYBL2 regulates the resistance of cervical cancer cells to paclitaxel via miR-665-dependent regulation of EGFR. Drug Dev. Res. 82 (8), 1193–1205. 10.1002/ddr.21834 34046939

[B29] Fu L. Zhang D. Yi N. Cao Y. Wei Y. Wang W. (2022). Circular RNA circPBX3 promotes cisplatin resistance of ovarian cancer cells via interacting with IGF2BP2 to stabilize ATP7A mRNA expression. Hum. Cell. 35 (5), 1560–1576. 10.1007/s13577-022-00748-8 35907138

[B30] Gao X. Tian X. Huang Y. Fang R. Wang G. (2022). Role of circular RNA in myocardial ischemia and ageing-related diseases. Cytokine Growth Factor Rev. 65, 1–11. 10.1016/j.cytogfr.2022.04.005 35561533

[B31] Geng X. Jia Y. Zhang Y. Shi L. Li Q. Zang A. (2020). Circular RNA: Biogenesis, degradation, functions and potential roles in mediating resistance to anticarcinogens. Epigenomics 12 (3), 267–283. 10.2217/epi-2019-0295 31808351

[B32] Gjorgoska M. Rizner T. L. (2022). Integration of androgen hormones in endometrial cancer biology. Trends Endocrinol. Metab. 33 (9), 639–651. 10.1016/j.tem.2022.06.001 35879182

[B33] Goldsbury D. E. Yap S. Weber M. F. Veerman L. Rankin N. Banks E. (2018). Health services costs for cancer care in Australia: Estimates from the 45 and up Study. PLoS One 13 (7), 0201552. 10.1371/journal.pone.0201552 PMC6066250 30059534

[B34] Gordhandas S. Zammarrelli W. A. Rios-Doria E. V. Green A. K. Makker V. (2023). Current evidence-based systemic therapy for advanced and recurrent endometrial cancer. J. Natl. Compr. Canc Netw. 21 (2), 217–226. 10.6004/jnccn.2022.7254 36791759 PMC10361357

[B35] Guo J. Chen M. Ai G. Mao W. Li H. Zhou J. (2019). Hsa_circ_0023404 enhances cervical cancer metastasis and chemoresistance through VEGFA and autophagy signaling by sponging miR-5047. Biomed. Pharmacother. 115, 108957. 10.1016/j.biopha.2019.108957 31082770

[B36] Guo M. Li S. Zhao X. Yuan Y. Zhang B. Guan Y. (2020). Knockdown of circular RNA Hsa_circ_0000714 can regulate RAB17 by sponging miR-370-3p to reduce paclitaxel resistance of ovarian cancer through CDK6/RB pathway. Onco Targets Ther. 13, 13211–13224. 10.2147/OTT.S285153 33380810 PMC7769200

[B37] Guo W. Dong W. Li M. Shen Y. (2019). Mitochondria P-glycoprotein confers paclitaxel resistance on ovarian cancer cells. Onco Targets Ther. 12, 3881–3891. 10.2147/OTT.S193433 31190887 PMC6529025

[B38] Huang C. Qin L. Chen S. Huang Q. (2023). CircSETDB1 contributes to paclitaxel resistance of ovarian cancer cells by sponging miR-508-3p and regulating ABCC1 expression. Anticancer Drugs 34 (3), 395–404. 10.1097/CAD.0000000000001465 36729852

[B39] Huang Y. Zhu Q. (2021). Mechanisms regulating abnormal circular RNA biogenesis in cancer. Cancers (Basel) 13 (16), 4185. 10.3390/cancers13164185 34439339 PMC8391333

[B40] Jamieson A. McAlpine J. N. (2023). Molecular profiling of endometrial cancer from TCGA to clinical practice. J. Natl. Compr. Canc Netw. 21 (2), 210–216. 10.6004/jnccn.2022.7096 36791751

[B41] Kalampokas E. Giannis G. Kalampokas T. Papathanasiou A. A. Mitsopoulou D. Tsironi E. (2022). Current approaches to the management of patients with endometrial cancer. Cancers (Basel) 14 (18), 4500. 10.3390/cancers14184500 36139659 PMC9497194

[B42] Kamazawa S. Kigawa J. Kanamori Y. Itamochi H. Sato S. Iba T. (2002). Multidrug resistance gene-1 is a useful predictor of Paclitaxel-based chemotherapy for patients with ovarian cancer. Gynecol. Oncol. 86 (2), 171–176. 10.1006/gyno.2002.6738 12144824

[B43] Karami Fath M. Azargoonjahromi A. Kiani A. Jalalifar F. Osati P. Akbari Oryani M. (2022). The role of epigenetic modifications in drug resistance and treatment of breast cancer. Cell. Mol. Biol. Lett. 27 (1), 52. 10.1186/s11658-022-00344-6 35764927 PMC9238060

[B44] Kim E. Kim Y. K. Lee S. V. (2021). Emerging functions of circular RNA in aging. Trends Genet. 37, 819–829. 10.1016/j.tig.2021.04.014 34016449

[B45] Kim W. R. Park E. G. Lee D. H. Lee Y. J. Bae W. H. Kim H. S. (2023). The tumorigenic role of circular RNA-MicroRNA Axis in cancer. Int. J. Mol. Sci. 24 (3), 3050. 10.3390/ijms24033050 36769372 PMC9917898

[B46] Lee K. H. Kim S. Lee S. W. (2022). Pros and cons of *in vitro* methods for circular RNA preparation. Int. J. Mol. Sci. 23 (21), 13247. 10.3390/ijms232113247 36362032 PMC9654983

[B47] Li F. Zheng Z. Chen W. Li D. Zhang H. Zhu Y. (2023). Regulation of cisplatin resistance in bladder cancer by epigenetic mechanisms. Drug Resist Updat 68, 100938. 10.1016/j.drup.2023.100938 36774746

[B48] Li H. Luo F. Jiang X. Zhang W. Xiang T. Pan Q. (2022). CircITGB6 promotes ovarian cancer cisplatin resistance by resetting tumor-associated macrophage polarization toward the M2 phenotype. J. Immunother. Cancer 10 (3), e004029. 10.1136/jitc-2021-004029 35277458 PMC8919471

[B49] Li J. Li X. Guo Q. (2022). Drug resistance in cancers: A free pass for bullying. Cells 11 (21), 3383. 10.3390/cells11213383 36359776 PMC9654341

[B50] Li J. Zhang Z. Hu Y. Wei Q. Shao X. (2022). Circ_0039569 contributes to the paclitaxel resistance of endometrial cancer via targeting miR-1271-5p/PHF6 pathway. Anticancer Drugs 33 (9), 883–892. 10.1097/CAD.0000000000001377 36136988

[B51] Li J. Zhu L. Kwok H. F. (2023). Nanotechnology-based approaches overcome lung cancer drug resistance through diagnosis and treatment. Drug Resist Updat 66, 100904. 10.1016/j.drup.2022.100904 36462375

[B52] Li M. Cai J. Han X. Ren Y. (2020). Downregulation of circNRIP1 suppresses the paclitaxel resistance of ovarian cancer via regulating the miR-211-5p/HOXC8 Axis. Cancer Manag. Res. 12, 9159–9171. 10.2147/CMAR.S268872 33061608 PMC7532313

[B53] Li X. Li M. Huang M. Lin Q. Fang Q. Liu J. (2022). The multi-molecular mechanisms of tumor-targeted drug resistance in precision medicine. Biomed. Pharmacother. 150, 113064. 10.1016/j.biopha.2022.113064 35658234

[B54] Liang Y. X. Zhang L. L. Yang L. (2022). circANKRD17(has_circ_0007883) confers paclitaxel resistance of ovarian cancer via interacting with FUS to stabilize FOXR2. Mol. Cell. Biochem. 478, 835–850. 10.1007/s11010-022-04548-4 36107285

[B55] Liu X. Zhang Y. Zhou S. Dain L. Mei L. Zhu G. (2022). Circular RNA: An emerging frontier in RNA therapeutic targets, RNA therapeutics, and mRNA vaccines. J. Control Release 348, 84–94. 10.1016/j.jconrel.2022.05.043 35649485 PMC9644292

[B56] Liu Z. Zou H. Dang Q. Xu H. Liu L. Zhang Y. (2022). Biological and pharmacological roles of m(6)A modifications in cancer drug resistance. Mol. Cancer 21 (1), 220. 10.1186/s12943-022-01680-z 36517820 PMC9749187

[B57] Lõhmussaar K. Boretto M. Clevers H. (2020). Human-derived model systems in gynecological cancer research. Trends Cancer 6 (12), 1031–1043. 10.1016/j.trecan.2020.07.007 32855097

[B58] Lugones Y. Loren P. Salazar L. A. (2022). Cisplatin resistance: Genetic and epigenetic factors involved. Biomolecules 12 (10), 1365. 10.3390/biom12101365 36291573 PMC9599500

[B59] Luo M. Deng X. Chen Z. Hu Y. (2023). Circular RNA circPOFUT1 enhances malignant phenotypes and autophagy-associated chemoresistance via sequestrating miR-488-3p to activate the PLAG1-ATG12 axis in gastric cancer. Cell. Death Dis. 14 (1), 10. 10.1038/s41419-022-05506-0 36624091 PMC9829716

[B60] Luo Y. Gui R. (2020). Circulating exosomal circFoxp1 confers cisplatin resistance in epithelial ovarian cancer cells. J. Gynecol. Oncol. 31 (5), e75. 10.3802/jgo.2020.31.e75 32808501 PMC7440976

[B61] Meng X. Xiao W. Sun J. Li W. Yuan H. Yu T. (2023). CircPTK2/PABPC1/SETDB1 axis promotes EMT-mediated tumor metastasis and gemcitabine resistance in bladder cancer. Cancer Lett. 554, 216023. 10.1016/j.canlet.2022.216023 36436682

[B62] Ming H. Li B. Jiang J. Qin S. Nice E. C. He W. (2023). Protein degradation: Expanding the toolbox to restrain cancer drug resistance. J. Hematol. Oncol. 16 (1), 6. 10.1186/s13045-023-01398-5 36694209 PMC9872387

[B63] Nielsen A. F. Bindereif A. Bozzoni I. Hanan M. Hansen T. B. Irimia M. (2022). Best practice standards for circular RNA research. Nat. Methods 19, 1208–1220. 10.1038/s41592-022-01487-2 35618955 PMC9759028

[B64] Ortiz M. Wabel E. Mitchell K. Horibata S. (2022). Mechanisms of chemotherapy resistance in ovarian cancer. Cancer Drug Resist 5 (2), 304–316. 10.20517/cdr.2021.147 35800369 PMC9255249

[B65] Pang K. Shi Z. D. Wei L. Y. Dong Y. Ma Y. Y. Wang W. (2023). Research progress of therapeutic effects and drug resistance of immunotherapy based on PD-1/PD-L1 blockade. Drug Resist Updat 66, 100907. 10.1016/j.drup.2022.100907 36527888

[B66] Parma B. Wurdak H. Ceppi P. (2022). Harnessing mitochondrial metabolism and drug resistance in non-small cell lung cancer and beyond by blocking heat-shock proteins. Drug Resist Updat 65, 100888. 10.1016/j.drup.2022.100888 36332495

[B67] Passarello K. Kurian S. Villanueva V. (2019). Endometrial cancer: An overview of pathophysiology, management, and care. Semin. Oncol. Nurs. 35 (2), 157–165. 10.1016/j.soncn.2019.02.002 30867105

[B68] Pastwińska J. Karaś K. Karwaciak I. Ratajewski M. (2022). Targeting EGFR in melanoma - the sea of possibilities to overcome drug resistance. Biochim. Biophys. Acta Rev. Cancer 1877 (4), 188754. 10.1016/j.bbcan.2022.188754 35772580

[B69] Rahangdale L. Mungo C. O'Connor S. Chibwesha C. J. Brewer N. T. (2022). Human papillomavirus vaccination and cervical cancer risk. Bmj 379, e070115. 10.1136/bmj-2022-070115 36521855

[B70] Rao Y. Zhang W. Li D. Li X. Ma Y. Qu P. (2022). Circ TYMP1 inhibits carcinogenesis and cisplatin resistance in ovarian cancer by reducing smad2/3 phosphorylation via a MicroRNA-182a-3p/TGF1B Axis. Contrast Media Mol. Imaging 2022, 1032557. 10.1155/2022/1032557 36072623 PMC9398837

[B71] Reich O. Regauer S. (2023). Elimination of reserve cells for prevention of HPV-associated cervical cancer. Virus Res. 329, 199068. 10.1016/j.virusres.2023.199068 36854360 PMC10194256

[B72] Ren L. Jiang Q. Mo L. Tan L. Dong Q. Meng L. (2022). Mechanisms of circular RNA degradation. Commun. Biol. 5 (1), 1355. 10.1038/s42003-022-04262-3 36494488 PMC9734648

[B73] Rimel B. J. Kunos C. A. Macioce N. Temkin S. M. (2022). Current gaps and opportunities in screening, prevention, and treatment of cervical cancer. Cancer 128 (23), 4063–4073. 10.1002/cncr.34487 36239009

[B74] Romani A. M. P. (2022). Cisplatin in cancer treatment. Biochem. Pharmacol. 206, 115323. 10.1016/j.bcp.2022.115323 36368406

[B75] Rubinstein M. Shen S. Monk B. J. Tan D. S. P. Nogueira-Rodrigues A. Aoki D. (2022). Looking beyond carboplatin and paclitaxel for the treatment of advanced/recurrent endometrial cancer. Gynecol. Oncol. 167 (3), 540–546. 10.1016/j.ygyno.2022.10.012 36280455 PMC10373231

[B76] Sabeena S. (2023). Role of noncoding RNAs with emphasis on long noncoding RNAs as cervical cancer biomarkers. J. Med. Virol. 95 (2), e28525. 10.1002/jmv.28525 36702772

[B77] Sánchez-Marín D. Trujano-Camacho S. Pérez-Plasencia C. De León D. C. Campos-Parra A. D. (2022). LncRNAs driving feedback loops to boost drug resistance: Sinuous pathways in cancer. Cancer Lett. 543, 215763. 10.1016/j.canlet.2022.215763 35680071

[B78] Shao T. Pan Y. H. Xiong X. D. (2021). Circular RNA: An important player with multiple facets to regulate its parental gene expression. Mol. Ther. Nucleic Acids 23, 369–376. 10.1016/j.omtn.2020.11.008 33425494 PMC7779830

[B79] Sheng H. Wang X. (2023). Knockdown of circ-PIP5K1A overcomes resistance to cisplatin in ovarian cancer by miR-942-5p/NFIB axis. Anticancer Drugs 34 (2), 214–226. 10.1097/CAD.0000000000001406 36730637

[B80] Sinha T. Panigrahi C. Das D. Chandra Panda A. (2021). Circular RNA translation, a path to hidden proteome. Wiley Interdiscip. Rev. RNA 13, e1685. 10.1002/wrna.1685 34342387 PMC7613019

[B81] Sivars L. Palsdottir K. Crona Guterstam Y. Falconer H. Hellman K. Tham E. (2022). The current status of cell-free human papillomavirus DNA as a biomarker in cervical cancer and other HPV-associated tumors: A review. Int. J. Cancer 152, 2232–2242. 10.1002/ijc.34333 36274628

[B82] Smith E. R. Wang J. Q. Yang D. H. Xu X. X. (2022). Paclitaxel resistance related to nuclear envelope structural sturdiness. Drug Resist Updat 65, 100881. 10.1016/j.drup.2022.100881 36368286

[B83] Sun Q. Wang L. Zhang C. Hong Z. Han Z. (2022). Cervical cancer heterogeneity: A constant battle against viruses and drugs. Biomark. Res. 10 (1), 85. 10.1186/s40364-022-00428-7 36397138 PMC9670454

[B84] Sun Y. Li X. Chen A. Shi W. Wang L. Yi R. (2019). circPIP5K1A serves as a competitive endogenous RNA contributing to ovarian cancer progression via regulation of miR-661/IGFBP5 signaling. J. Cell. Biochem. 120 (12), 19406–19414. 10.1002/jcb.29055 31452245

[B85] Sung H. Ferlay J. Siegel R. L. Laversanne M. Soerjomataram I. Jemal A. (2021). Global cancer statistics 2020: GLOBOCAN estimates of incidence and mortality worldwide for 36 cancers in 185 countries. CA Cancer J. Clin. 71 (3), 209–249. 10.3322/caac.21660 33538338

[B86] Tang C. Livingston M. J. Safirstein R. Dong Z. (2023). Cisplatin nephrotoxicity: New insights and therapeutic implications. Nat. Rev. Nephrol. 19 (1), 53–72. 10.1038/s41581-022-00631-7 36229672

[B87] Tang M. Lv Y. (2021). The role of *N<sup>6</sup>* -methyladenosine modified circular RNA in pathophysiological processes. Int. J. Biol. Sci. 17 (9), 2262–2277. 10.7150/ijbs.60131 34239354 PMC8241720

[B88] Tau S. Miller T. W. (2023). The role of cancer cell bioenergetics in dormancy and drug resistance. Cancer Metastasis Rev. 42, 87–98. 10.1007/s10555-023-10081-7 36696004 PMC10233409

[B89] Terp S. K. Stoico M. P. Dybkær K. Pedersen I. S. (2023). Early diagnosis of ovarian cancer based on methylation profiles in peripheral blood cell-free DNA: A systematic review. Clin. Epigenetics 15 (1), 24. 10.1186/s13148-023-01440-w 36788585 PMC9926627

[B90] Tronconi F. Nero C. Giudice E. Salutari V. Musacchio L. Ricci C. (2022). Advanced and recurrent endometrial cancer: State of the art and future perspectives. Crit. Rev. Oncol. Hematol. 180, 103851. 10.1016/j.critrevonc.2022.103851 36257537

[B91] Tsvetkova D. Ivanova S. (2022). Application of approved cisplatin derivatives in combination therapy against different cancer diseases. Molecules 27 (8), 2466. 10.3390/molecules27082466 35458666 PMC9031877

[B92] Vaghari-Tabari M. Hassanpour P. Sadeghsoltani F. Malakoti F. Alemi F. Qujeq D. (2022). CRISPR/Cas9 gene editing: A new approach for overcoming drug resistance in cancer. Cell. Mol. Biol. Lett. 27 (1), 49. 10.1186/s11658-022-00348-2 35715750 PMC9204876

[B93] Wang Q. Shen X. Chen G. Du J. (2022). Drug resistance in colorectal cancer: From mechanism to clinic. Cancers (Basel) 14 (12), 2928. 10.3390/cancers14122928 35740594 PMC9221177

[B94] Wang X. Jiang W. Du Y. Zhu D. Zhang J. Fang C. (2022). Targeting feedback activation of signaling transduction pathways to overcome drug resistance in cancer. Drug Resist Updat 65, 100884. 10.1016/j.drup.2022.100884 36252360

[B95] Wang X. Zhou Y. Wang D. Wang Y. Zhou Z. Ma X. (2023). Cisplatin-induced ototoxicity: From signaling network to therapeutic targets. Biomed. Pharmacother. 157, 114045. 10.1016/j.biopha.2022.114045 36455457

[B96] Wang Y. Wu C. Du Y. Li Z. Li M. Hou P. (2022). Expanding uncapped translation and emerging function of circular RNA in carcinomas and noncarcinomas. Mol. Cancer 21 (1), 13. 10.1186/s12943-021-01484-7 34996480 PMC8740365

[B97] Webb P. M. Jordan S. J. (2017). Epidemiology of epithelial ovarian cancer. Best. Pract. Res. Clin. Obstet. Gynaecol. 41, 3–14. 10.1016/j.bpobgyn.2016.08.006 27743768

[B98] Wei L. He W. Zhao H. Zhao P. (2022). Circ_0026123 promotes cisplatin resistance and progression of ovarian cancer by upregulating RAB1A through sequestering miR-543. Anticancer Drugs 33 (10), 1069–1080. 10.1097/CAD.0000000000001373 36255068

[B99] Wei S. Qi L. Wang L. (2021). Overexpression of circ_CELSR1 facilitates paclitaxel resistance of ovarian cancer by regulating miR-149-5p/SIK2 axis. Anticancer Drugs 32 (5), 496–507. 10.1097/CAD.0000000000001058 33735118

[B100] Wen S. Y. Qadir J. Yang B. B. (2022). Circular RNA translation: Novel protein isoforms and clinical significance. Trends Mol. Med. 28 (5), 405–420. 10.1016/j.molmed.2022.03.003 35379558

[B101] Wu H. Zhao X. Wang J. Jiang X. Cheng Y. He Y. (2021). Circular RNA CDR1as alleviates cisplatin-based chemoresistance by suppressing MiR-1299 in ovarian cancer. Front. Genet. 12, 815448. 10.3389/fgene.2021.815448 35154259 PMC8826532

[B102] Wu J. Guo X. Wen Y. Huang S. Yuan X. Tang L. (2021). N6-Methyladenosine modification opens a new chapter in circular RNA biology. Front. Cell. Dev. Biol. 9, 709299. 10.3389/fcell.2021.709299 34368159 PMC8342999

[B103] Wu P. Qin J. Liu L. Tan W. Lei L. Zhu J. (2022). circEPSTI1 promotes tumor progression and cisplatin resistance via upregulating MSH2 in cervical cancer. Aging (Albany NY) 14 (13), 5406–5416. 10.18632/aging.204152 35779530 PMC9320557

[B104] Xia B. Zhao Z. Wu Y. Wang Y. Zhao Y. Wang J. (2020). Circular RNA circTNPO3 regulates paclitaxel resistance of ovarian cancer cells by miR-1299/NEK2 signaling pathway. Mol. Ther. Nucleic Acids 21, 780–791. 10.1016/j.omtn.2020.06.002 32791450 PMC7419276

[B105] Xu T. He B. Sun H. Xiong M. Nie J. Wang S. (2022). Novel insights into the interaction between N6-methyladenosine modification and circular RNA. Mol. Ther. Nucleic Acids 27, 824–837. 10.1016/j.omtn.2022.01.007 35141044 PMC8807973

[B106] Xu Y. Jiang Z. Chen X. (2022). Mechanisms underlying paclitaxel-induced neuropathic pain: Channels, inflammation and immune regulations. Eur. J. Pharmacol. 933, 175288. 10.1016/j.ejphar.2022.175288 36122757

[B107] Yang C. Mai Z. Liu C. Yin S. Cai Y. Xia C. (2022). Natural products in preventing tumor drug resistance and related signaling pathways. Molecules 27 (11), 3513. 10.3390/molecules27113513 35684449 PMC9181879

[B108] Yang Q. Xu J. Gu J. Shi H. Zhang J. Zhang J. (2022). Extracellular vesicles in cancer drug resistance: Roles, mechanisms, and implications. Adv. Sci. (Weinh) 9, 2201609. 10.1002/advs.202201609 36253096 PMC9731723

[B109] Yang R. Yi M. Xiang B. (2022). Novel insights on lipid metabolism alterations in drug resistance in cancer. Front. Cell. Dev. Biol. 10, 875318. 10.3389/fcell.2022.875318 35646898 PMC9136290

[B110] Ye L. Yao X. Xu B. Chen W. Lou H. Tong X. (2023). RNA epigenetic modifications in ovarian cancer: The changes, chances, and challenges. Wiley Interdiscip. Rev. RNA 2023, e1784. 10.1002/wrna.1784 36811232

[B111] Yi H. Han Y. Li Q. Wang X. Xiong L. Li S. (2022). Circular RNA circ_0004488 increases cervical cancer paclitaxel resistance via the miR-136/MEX3C signaling pathway. J. Oncol. 2022, 5435333. 10.1155/2022/5435333 36439901 PMC9691333

[B112] Yi H. Han Y. Li S. (2022). Oncogenic circular RNA circ_0007534 contributes to paclitaxel resistance in endometrial cancer by sponging miR-625 and promoting ZEB2 expression. Front. Oncol. 12, 985470. 10.3389/fonc.2022.985470 35992812 PMC9386306

[B113] You J. Han Y. Qiao H. Han Y. Lu X. Lu Y. (2022). Hsa_circ_0063804 enhances ovarian cancer cells proliferation and resistance to cisplatin by targeting miR-1276/CLU axis. Aging (Albany NY) 14 (11), 4699–4713. 10.18632/aging.203474 35687899 PMC9217714

[B114] Yu R. Matthews B. J. Beavis A. L. (2022). The role of sentinel lymph node mapping in high-grade endometrial cancer. Curr. Treat. Options Oncol. 23 (10), 1339–1352. 10.1007/s11864-022-00999-5 35980519

[B115] Yuan S. Zheng P. Sun X. Zeng J. Cao W. Gao W. (2021). Hsa_Circ_0001860 promotes Smad7 to enhance MPA resistance in endometrial cancer via miR-520h. Front. Cell. Dev. Biol. 9, 738189. 10.3389/fcell.2021.738189 34912799 PMC8666979

[B116] Yuan Y. Zhang X. Fan X. Peng Y. Jin Z. (2022). The emerging roles of circular RNA-mediated autophagy in tumorigenesis and cancer progression. Cell. Death Discov. 8 (1), 385. 10.1038/s41420-022-01172-5 36104321 PMC9474543

[B117] Zhang C. Ju J. Wu X. Yang J. Yang Q. Liu C. (2021). *Tripterygium wilfordii* polyglycoside ameliorated TNBS-induced colitis in rats via regulating Th17/treg balance in intestinal mucosa. Mol. Ther. Nucleic Acids 23, 1243–1255. 10.2147/JIR.S293961 33833546 PMC8021269

[B118] Zhang S. Cheng J. Quan C. Wen H. Feng Z. Hu Q. (2020). circCELSR1 (hsa_circ_0063809) contributes to paclitaxel resistance of ovarian cancer cells by regulating FOXR2 expression via miR-1252. Mol. Ther. Nucleic Acids 19, 718–730. 10.1016/j.omtn.2019.12.005 31945729 PMC6965731

[B119] Zhao B. Gu Z. Zhang Y. Li Z. Cheng L. (2022). Starch-based carriers of paclitaxel: A systematic review of carriers, interactions, and mechanisms. Carbohydr. Polym. 291, 119628. 10.1016/j.carbpol.2022.119628 35698420

[B120] Zhao L. Guo H. Chen X. Zhang W. He Q. Ding L. (2022). Tackling drug resistance in ovarian cancer with epigenetic targeted drugs. Eur. J. Pharmacol. 927, 175071. 10.1016/j.ejphar.2022.175071 35636522

[B121] Zhao Y. Lan Y. Chi Y. Yang B. Ren C. (2022). Downregulation of circ-cep128 enhances the paclitaxel sensitivity of cervical cancer through regulating miR-432-5p/MCL1. Biochem. Genet. 60, 2346–2363. 10.1007/s10528-022-10201-y 35391656

[B122] Zhao Z. Ji M. Wang Q. He N. Li Y. (2019). Circular RNA Cdr1as upregulates SCAI to suppress cisplatin resistance in ovarian cancer via miR-1270 suppression. Mol. Ther. Nucleic Acids 18, 24–33. 10.1016/j.omtn.2019.07.012 31479922 PMC6726918

[B123] Zheng Y. Li Z. Yang S. Wang Y. Luan Z. (2020). CircEXOC6B suppresses the proliferation and motility and sensitizes ovarian cancer cells to paclitaxel through miR-376c-3p/FOXO3 Axis. Cancer Biother Radiopharm. 37, 802–814. 10.1089/cbr.2020.3739 33006481

[B124] Zhong G. Zhao Q. Chen Z. Yao T. (2023). TGF-beta signaling promotes cervical cancer metastasis via CDR1as. Mol. Cancer 22 (1), 66. 10.1186/s12943-023-01743-9 37004067 PMC10064584

[B125] Zhou M. Xiao M. S. Li Z. Huang C. (2021). New progresses of circular RNA biology: From nuclear export to degradation. RNA Biol. 18 (10), 1–9. 10.1080/15476286.2020.1853977 PMC8489929 33241761

[B126] Zhou W. Y. Cai Z. R. Liu J. Wang D. S. Ju H. Q. Xu R. H. (2020). Circular RNA: Metabolism, functions and interactions with proteins. Mol. Cancer 19 (1), 172. 10.1186/s12943-020-01286-3 33317550 PMC7734744

[B127] Zhou X. Lin J. Wang F. Chen X. Zhang Y. Hu Z. (2022). Circular RNA-regulated autophagy is involved in cancer progression. Front. Cell. Dev. Biol. 10, 961983. 10.3389/fcell.2022.961983 36187468 PMC9515439

[B128] Zhu J. Luo J. E. Chen Y. Wu Q. (2021). Circ_0061140 knockdown inhibits tumorigenesis and improves PTX sensitivity by regulating miR-136/CBX2 axis in ovarian cancer. J. Ovarian Res. 14 (1), 136. 10.1186/s13048-021-00888-9 34649611 PMC8518226

[B129] Zwimpfer T. A. Tal O. Geissler F. Coelho R. Rimmer N. Jacob F. (2023). Low grade serous ovarian cancer - a rare disease with increasing therapeutic options. Cancer Treat. Rev. 112, 102497. 10.1016/j.ctrv.2022.102497 36525716

